# The Role of Exercise-Based Cardiac Rehabilitation After Myocardial Infarction on Cholesterol Transfer to HDL

**DOI:** 10.3390/ijms26010419

**Published:** 2025-01-06

**Authors:** Jose C. Nicolau, Talia F. Dalcoquio, Roberto R. Giraldez, Fatima R. Freitas, Andre M. Nicolau, Remo H. M. Furtado, Thauany M. Tavoni, Luciano M. Baracioli, Felipe G. Lima, Aline G. Ferrari, Maria U. P. B. Rondon, Rocio Salsoso, Maria J. N. N. Alves, Flavia B. B. Arantes, Mayara A. Santos, Leandro S. Alves, Carlos E. Negrao, Raul C. Maranhão

**Affiliations:** 1 Instituto do Coracao (InCor) Hospital das Clinicas, Faculdade de Medicina, Universidade de Sao Paulo, Sao Paulo 05508-220, SP, Brazil; roberto.giraldez1@gmail.com (R.R.G.); andremoreiranicolau@gmail.com (A.M.N.); lumobaracioli@gmail.com (L.M.B.); felipe.gallego@hc.fm.usp.br (F.G.L.); silva.profleandro@gmail.com (L.S.A.); carlos.negrao@hc.fm.usp.br (C.E.N.); 2 Hospital Sírio-Libanês, São Paulo 01308-050, SP, Brazil; 3 Escola de Educacao Fisica e Esporte, Universidade de Sao Paulo, São Paulo 05508-060, SP, Brazil; 4 Instituto de Biomedicina de Sevilla, Hospital Universitario Virgen del Rocío/Consejo Superior de Investigaciones Científicas/Universidad de Sevilla, 41013 Sevilla, Spain; 5 Faculdade de Ciencias Farmaceuticas, Universidade de Sao Paulo, São Paulo 05508-000, SP, Brazil

**Keywords:** acute myocardial infarction, HDL functionality, supervised exercise, cardiac rehabilitation

## Abstract

High-density lipoprotein (HDL) is associated with decreased incidence of cardiovascular events, and its functionality also influences prognosis. Exercise is an important tool to improve prognosis in the post-infarction (MI) population, but the role of exercise on HDL functionality is poorly understood. Sixty-two patients with acute MI were randomized in a supervised exercise program for 12–14 weeks (exercise group—EG) or a control group (CG). The main objective of the study was to analyze the role of exercise on esterified cholesterol (EC) and unesterified cholesterol (UC) transfer to HDL. For the total population, the baseline mean rate of EC transfer to HDL was 2.53 ± 0.83 and at the end of follow-up, it was 2.74 ± 0.64 (*p* = 0.03). The figures for UC were, respectively, 4.08 ± 1.2 and 4.4 ± 1.06 (*p* = 0.02). The difference (follow-up minus baseline) for EC was 0.15 ± 0.84 for the control group and 0.27 ± 0.69 for the exercise group (*p* = 0.53); for UC, the figures were 0.28 ± 1.14 and 0.35 ± 0.96 (*p* = 0.80), respectively, for the control and exercise groups. In post-MI patients, 12–14 weeks of supervised exercise did not improve HDL functionality.

## 1. Introduction

Myocardial infarction (MI) is a leading cause of morbidity and mortality globally, profoundly impacting patients’ quality of life and functional capacity [1,2,3]. Following an MI, the body undergoes significant biochemical and physiological changes, including changes in lipid metabolism [4]. High-density lipoprotein (HDL) is well recognized for its atheroprotective properties, primarily mediated through its role in reverse cholesterol transport and esterification of cholesterol in the plasma, along with several other beneficial effects, such as its anti-inflammatory actions [5,6]. Analyzing approximately 3000 individuals free from cardiovascular diseases, Rohatgi et al. [7] found a significant inverse association between cholesterol efflux capacity and cardiovascular events in a median follow-up of 9.4 years. However, myocardial infarction can impair HDL functionality, contributing to a higher risk of recurrent cardiovascular events, including short- and long-term mortality [8,9,10,11,12].

Besides receiving unesterified cholesterol (UC) from the peripheral cells in the so-called reverse cholesterol transport, in the intravascular compartment, HDL continuously receives UC and esterified cholesterol (EC) from the apo B-containing lipoproteins. This process changes HDL composition and function, is fundamental for cholesterol homeostasis in the plasma, and is also part of the reverse cholesterol transport. UC received by HDL is esterified by lecithin–cholesterol-acyl-transferase (LCAT) with apo A-I and may be transferred to apo B-containing lipoproteins [13]

Despite the fact that the high risk of recurrent ischemic events post-MI is not fully understood [14,15], exercise-based cardiac rehabilitation (CR) has been shown to improve cardiovascular outcomes by enhancing physical fitness, reducing symptoms, improving autonomic regulation, endothelial function, and inflammatory activity, and leading to a decrease in the likelihood of future cardiovascular events [16,17,18]. Indeed, cardiorespiratory fitness is a strong quantitative predictor of all-cause mortality [19], but it is important to note that a specific dose–response relationship between the extent and duration of exercise, and the reduction in cardiovascular risk and mortality, remains unclear [20]. So, even though moderate exercise-based CR is a cornerstone of post-MI treatment, with well-documented benefits that extend beyond improved cardiovascular endurance, the specific mechanisms by which exercise-based CR influences lipid metabolism, particularly HDL functionality, remain underexplored, with mixed evidence that regular aerobic exercise improves cholesterol efflux capacity [21,22]. Interestingly, in an extensive review analyzing the role of exercise training on cardiovascular health, there is no mention of HDL and its HDL functional aspects, which are increasingly perceived as more important than the single HDL–cholesterol (HDL-C) determination to evaluate cardiovascular risk [23].

As current therapeutic strategies for cardiovascular diseases increasingly emphasize the role of HDL function rather than merely HDL-C levels, it is critical to determine how exercise-based CR can influence this aspect of lipid metabolism [6,24].

In the present study, we hypothesized whether participation in a structured CR program could enhance HDL functionality in post-MI patients, thus providing further evidence for the performance of exercise training in reducing cardiovascular risk beyond traditional lipid management.

## 2. Results

**Figure 1:**
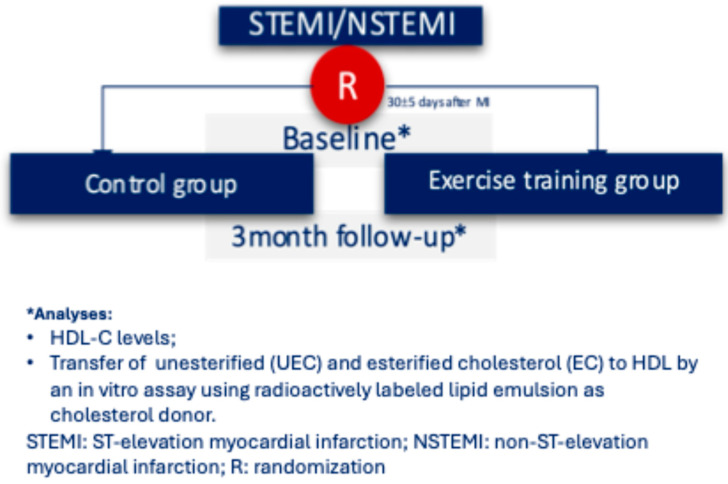
Flowchart of the study.

Figure 1 shows the flowchart of this study. Patients were randomized to control or exercise groups one month after the index MI and followed for 12–14 weeks afterward.

The clinical characteristics of the population are depicted in Table 1. The mean age of the population was 59 years, of which 74% were men, and the groups were similar regarding previous history, GRACE score, ST-elevation MI, renal function, troponin levels, left ventricular ejection fraction, and use of guideline-recommended therapies for patients after MI.

**Figure 2:**
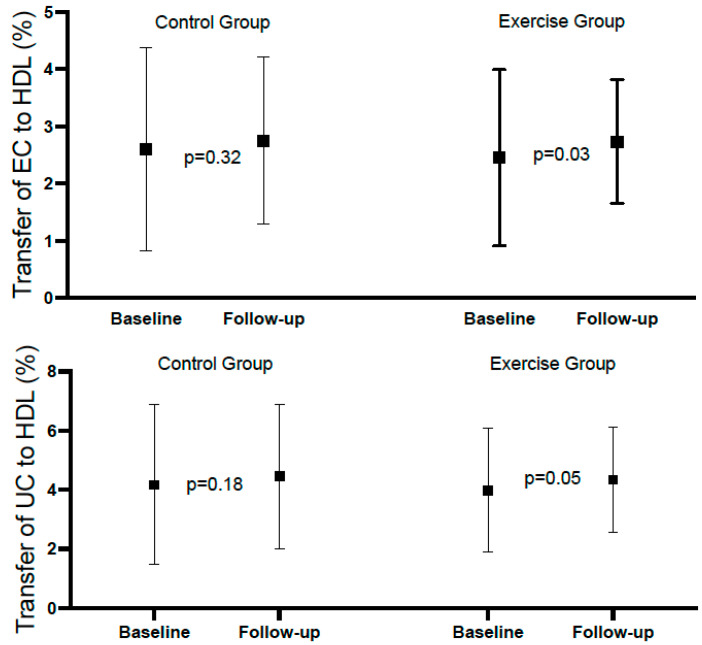
Forest plot of esterified and unesterified cholesterol to HDL in control and exercise group.

**Figure 3:**
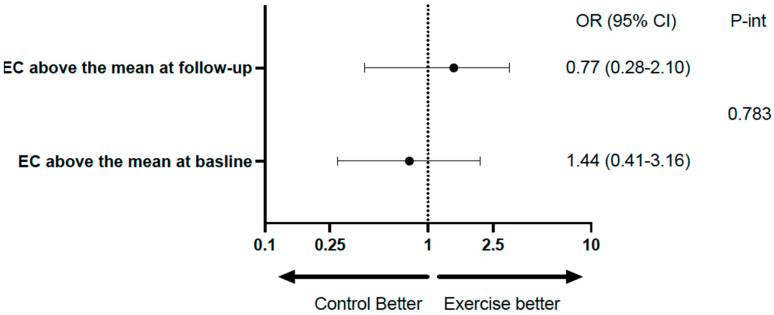
Forest plot illustrating the role of exercise on HDL transfer.

Table 2 shows the mean rates of transfer of EC and UC to HDL at baseline and at the end of follow-up in the total population, with both variables showing a significant increase at the end of follow-up. Table 3 and Figure 2 show the same analysis for each group. There was a significant increase in the exercise group, but not in the control group; however, the difference (follow-up minus baseline), despite being numerically higher in the exercise group, did not reach statistical significance. Considering categorical variables, there was no significant difference between the groups relative to EC, and the *p*-value for interaction was 0.783. Relative to UC, the percentages above the median at baseline and follow-up were identical in both groups (96.8%). For the comparison between baseline and follow-up of the percentages above the mean within groups, the differences did not reach statistical significance (Table 4 and Figure 3).

Regarding other lipid parameters, there was a significant increase in HDL–cholesterol and LDL–cholesterol in both groups during follow-up, with no significant differences between groups (Table 5). In a subgroup of 12 patients from the control group and 18 patients from the intervention group, the mean levels of apolipoprotein A1 at follow-up were 1.11 ± 0.19 and 1.13 ± 0.21, respectively (*p* = 0.844); in the same subgroup, the results for apolipoprotein B were 0.66 ± 0.04 and 0.69 ± 0.16, respectively (*p* = 0.598).

Table 6 shows the role of exercise on C-reactive protein (CRP) and peak VO_2_. There was a numerical decrease in the CRP after exercise, although the difference was not statistically significant (*p* = 0.06). Regarding peak VO_2_, there was a significant increase in the exercise group (*p* = 0.005) that was not observed in the control group, with a tendency (*p* = 0.092) toward significance for the comparison between groups.

## 3. Discussion

The present randomized study showed, in patients with acute MI, that the transfer of EC and UC to HDL decreased during the initial phase post-MI and increased significantly after 12–14 weeks, which was similar in patients submitted to a supervised exercise rehabilitation program or not.

A meta-analysis by Salzwedel et al. [25] analyzing the role of cardiac rehabilitation post-ACS (>50,000 patients) found a significant decrease in all-cause mortality in retrospective cohort studies (HR = 0.64) and prospective cohort studies (HR = 0.37), but the single randomized controlled trial that included >1800 patients showed neutral results (HR = 1.01).

Previous publications showed that exercise training increases the transfer of UC or EC to HDL in individuals with metabolic syndrome [26] and both normal-weight young and aged groups of healthy individuals [27]. Moreover, our group demonstrated that the EC transfer to HDL was reduced in patients on the fifth day after MI when compared to patients with <24 h after the onset of MI symptoms, whereas the UC transfer to HDL was similar between groups [9].

However, as pointed out by Ruiz-Ramie et al. [22] after an extensive review of the pertinent literature: “there is mixed evidence that regular aerobic exercise improves cholesterol efflux capacity”. These differences could be related to many factors, including exercise dose and participant characteristics, with few studies analyzing the issue in patients with myocardial infarction or acute coronary syndromes (ACS). In a retrospective study analyzing post-ACS patients participating in a 6-month outpatient cardiac rehabilitation program, Koba et al. [28] compared 57 patients who completed the program with 11 patients who dropped out of the program. Their main finding was that patients who completed the 6-month rehabilitation program experienced a significantly improved HDL-mediated efflux capacity. In a more recent publication from the same group with the same methodology (retrospective analysis), the authors compared a 5-month outpatient rehabilitation program with 15 patients who did not participate or dropped out from the program and showed a significant 9.4% increase in cholesterol efflux capacity, irrespective of the statin utilized [29].

On the other hand, Sarzynski et al. [30], analyzing the effects of exercise intensity and dose on HDL function, found an increase in HDL efflux capacity only in the high-amount/high-intensity intervention groups.

To the best of our knowledge, this is the first study analyzing in a randomized way a population with myocardial infarction, submitted or not, to a supervised exercise rehabilitation program, and did not find significant differences between the intervention and control groups, which could be related to the shorter time of training (12–14 weeks) in our population, in comparison with the above-cited studies (6 and 5 months), and/ or the intensity/mode of exercise we applied. Of note, in our population, despite not reaching statistical significance, the difference between baseline and follow-up for EC and UC was numerically higher in the intervention group, and the difference between baseline and follow-up for the transfer of EC to HDL showed a significant difference (*p* = 0.03) in the exercise group that was not observed in the control group.

### Impact of Exercise on Other Lipid Parameters, Inflammation, and Maximal Oxygen Consumption

Studies of the impact of regular exercise on plasma concentrations of HDL-C have provided conflicting results. These differences could be related to the utilization of different interventions, different types of patients, and different concomitant therapies, with the great majority of the publications analyzing the role of exercise in stable patients, and without control group/randomization [31,32].

The present study, analyzing post-MI patients, found a significant increase in the mean HDL-C in both, control and intervention groups, with no significant differences between groups. On the other hand, strict control of LDL-C is of paramount importance in this high-risk post-MI population, including those with MINOCA [33]. Our study showed a significant increase in LDL levels between baseline and the end of follow-up, in both groups, without significant differences between them. Considering that all but one patient was taking high-potency statin at baseline and everybody was taking statin at the end of follow-up, this increase could be related to the decrease in LDL during the initial phase of MI, and/or diet changes during follow-up.

Relatively to inflammation, metanalysis by Zheng et al. [34], analyzing 1250 healthy middle-aged and older individuals included in randomized controlled clinical trials, showed an important decrease in CRP levels (*p* = 0.0002) among the participants submitted to aerobic exercise, relative to those in the control group, with similar patterns observed for other inflammatory markers, such as interleukin 6. Specifically in secondary prevention, in a review by Lavie et al. [35], it is concluded that cardiac rehabilitation led to significant reductions in CRP levels, especially in overweight/obese patients. In the same direction, the present study, analyzing post-MI patients, showed a numerical decrease in CRP between follow-up and baseline levels in both exercise and control groups, with *p*-values of 0.06 and 0.72, respectively.

Regarding peak VO_2_, it has been accepted for decades that aerobic exercise is useful in improving this parameter [17], and a publication [36], analyzing 126 MI patients submitted to a cardiac rehabilitation program (no control group), found a significant increase in peak VO_2_ after three months of training in this high-risk population. Moreover, a meta-analysis with 12 studies analyzing the role of cardiac rehabilitation post-MI on peak VO_2_ compared 855 patients in the control group vs. 827 patients in the intervention group and demonstrated highly favorable results for the exercise group (*p* < 0.0001) [37]. The present study found a numerically higher improvement in peak VO_2_ in favor of the exercise group, with a tendency toward statistical significance (*p*-value = 0.092). It is important to note that, although not significant, the prevalence of current smokers in the exercise group was 83% higher in the exercise group, which could have influenced the results, given the fact that smoking negatively influences the VO_2_ max [38].

Our study has some limitations. First, with the rates of EC and UC transfer to HDL a secondary endpoint from the main study, the present results should be considered hypothesis-generating. Moreover, the relatively small sample size was probably underpowered for a more robust conclusion. Secondly, the period of supervised physical training (12 to 14 weeks) could have been too short to influence the analyzed parameters. Thirdly, other parameters that could have influenced the results, such as oxidative stress, were not obtained. Finally, although the rates of cholesterol transfer to HDL could be associated with protection from atherogenesis, it has not been ascertained whether changes in this surrogate endpoint can be translated into clinical benefits.

## 4. Materials and Methods

### 4.1. Study Design and Participants

This is a pre-specified secondary analysis of a prospective randomized study analyzing the role of exercise on platelet reactivity after myocardial infarction (NCT 02958657). Detailed methodology and main results have been published previously [39]. In short, patients with type I MI [40] who were not participating in regular exercise training prior to the index event, with a left ventricle ejection fraction of 45% or higher, a hematocrit level of between 32% and 52%, in the use of dual antiplatelet therapy and without planned coronary surgical revascularization or using oral anticoagulation, were prospectively randomized to a supervised training program (exercise group) or a control group. At hospital discharge, the use of proven therapies and lifestyle recommendations, including advice to engage in physical activity, were the same for patients allocated in both groups. All patients included in the trial had previously signed an informed consent and underwent a cardiopulmonary exercise test at the first visit (30 ± 5 days post-MI) and at the end of follow-up (12–14 weeks after the first visit).

Of the 65 patients included in the main publication [39], 62 had HDL-transfer evaluations at baseline and at the end of follow-up and were included in the present study (31 in each group, exercise or control). The other three patients did not have both HDL-transfer evaluations for logistic/technical issues.

### 4.2. Exercise Training Program

After the first study visit, patients randomized to the intervention group started a supervised exercise cardiac rehabilitation program at the Cardiovascular Rehabilitation and Exercise Physiology Unit of InCor/HCFMUSP. The exercise training program consisted of a minimum of two and a maximum of three sessions weekly (according to the patient’s availability). Each exercise session included 5 min of warm-up, 40 min of aerobic exercise on a cycle ergometer, 10 min of local strengthening exercises, and 5 min of cool down with stretching exercises. The aerobic exercise intensity was determined based on the heart rate that corresponded to the anaerobic threshold and 10% below the respiratory compensation point obtained in the initial cardiopulmonary exercise test. Patients with availability for training three times a week had a total follow-up of 12 weeks, and those with availability twice a week had a total follow-up of 14 weeks. With this methodology, the total volume of training was the same for all patients included in the exercise group. Blood samples were collected at baseline and the end of follow-up for the exercise group, and at baseline and after 12–14 weeks for the control group.

### 4.3. Laboratory Analyses

After 12 h of fasting, blood was collected to evaluate cholesterol transfer to HDL, among other parameters. The simultaneous transfer of esterified cholesterol (EC) and unesterified cholesterol (UC) from an artificial nanoparticle to HDL was measured in vitro, as described by Lo Prete et al. [13]. Briefly, donor lipid nanoparticle containing 3H-cholesteryl oleate and 14C-phosphatidylcholine or 3H-triolein and 14C-cholesterol was incubated with plasma under agitation for 1 h at 37 °C. After chemical precipitation of the nanoparticle and Apo B-containing lipo-proteins, the supernatant containing the HDL fraction was counted for radioactivity in a scintillation solution. Then, the percentage of each lipid transferred from the nanoparticle to HDL was calculated.

### 4.4. Study Purpose

The main purpose of the present study was to analyze the influence of exercise on the transfer of EC and UC to HDL. Secondary analyses included the influence of exercise on other lipid parameters and inflammation.

### 4.5. Statistical Analyses

Categorical variables are described as absolute numbers or percentages and the comparisons between groups utilized the chi-square test or Fisher’s exact test, as indicated. Continuous variables are described as mean and standard deviation (if with Gaussian distribution) or median and 25th–75th percentiles (if with non-Gaussian distribution). The Shapiro–Wilk test was used for normality evaluation. For the comparisons between groups, the Independent-samples Student’s *t*-test (Gaussian distribution) or the Mann–Whitney (non-Gaussian distribution) were utilized. The Paired Student’s *t*-test (Gaussian distributions) or Wilcoxon Signed Rank Test (non-Gaussian distributions) were used for the comparisons of HDL transfer between baseline and follow-up for the total population and for each group isolated. A binary logistic regression model was performed for the analyses of group interaction considering HDL transfer (as a categorical variable) for each group. The McNemar test for paired-sample proportions was utilized for the comparison between baseline and follow-up regarding the HDL transfer variables. All tests were two-tailed, and a value of *p* < 0.05 was considered statistically significant. The statistical software package used for statistical analysis was IBM SPSS 28.0 Statistics.

## 5. Conclusions

Among patients with myocardial infarction, our findings suggest that 12–14 weeks of supervised physical training do not significantly enhance EC and UC transfers to HDL compared to the control group. Taking all the patients together, there was a consistent increase in both EC and UC transfer to HDL during follow-up, suggesting a decrease in the rates of EC and UC transfer to HDL during the early phase of MI. In view of those results, it is tempting to suggest that future studies should test EC and UC transfers as markers of cardiac recovery post-MI.

## Figures and Tables

**Table 1:** Clinical characteristics of the population *.

Variables	Control Group (N = 31)	Exercise Group (N = 31)	*p*-Value
Age (mean ± SD)	58.5 ± 10.8	59.8 ± 9	0.60
Male Sex	74.2%	74.2%	1.00
History of HTN	67.7%	48.4%	0.12
History of DM	41.9%	22.6%	0.10
Previous Stroke	12.9%	6.5%	0.39
Previous MI	12.9%	12.9%	1.00
Previous CABG	6.5%	6.5%	1.00
Previous PCI	12.9%	12.9%	1.00
Current Smoking	19.4%	35.5%	0.15
STEMI	51.6%	64.5%	0.30
GRACE Score	130.3 ± 31.1	138.4 ± 24.3	0.30
LVEF	54.7 ± 7.3%	55.7 ± 6.3%	0.27
Creatinine (mg/dL—mean ± SD)	1.09 ± 0.2	1.01 ± 0.2	0.24
MDRD (mL/min—mean ± SD)	74.81 ± 23.5	82.87 ± 23.57	0.18
us-Troponin I (pg/mL—median 25th-75th percentile)	25 (2.9–50)	38.4 (11.8–50)	0.23
DAPT	100%	100%	1.00
Statin	96.8%	100%	0.31
Betablocker	66.7%	83.9%	0.14
ACEI or ARB	67.7%	77.4%	0.40

* At randomization (30 days post-index MI). All *p*-values = NS. ACEI: Angiotensin-converting Enzyme Inhibitor), ARB: Angiotensin Receptor Blocker; CABG: Coronary Artery Bypass Graft; DAPT: Dual Antiplatelet Therapy; DM; Diabetes Mellitus; GRACE: Global Registry of Acute Coronary Events; HTN: Hypertension; us-Troponin I: Ultra-sensitivity Troponin I; LVEF: Left Ventricular Ejection Fraction; MDRD: Modification of Diet in Renal Disease; MI: Myocardial Infarction; PCI: Percutaneous Coronary Intervention: STEMI: ST-Elevation Myocardial Infarction.

**Table 2:** Transfer of esterified (EC) and unesterified (UC) cholesterol to HDL at baseline and the end of follow-up for the total population.

Variables	Baseline	Follow-Up	*p*-Value
Transfer of EC to HDL	2.53 ± 0.83	2.74 ± 0.64	0.032
Transfer of UC to HDL	4.08 ± 1.2	4.40 ± 1.06	0.021

All values mean ± SD.

**Table 3:** Transfer of esterified cholesterol (EC) and unesterified cholesterol (UC) to HDL in the control and exercise groups.

Variables	Control Group (N = 31)	Exercise Group (N = 31)	*p*-Value
Transfer of EC to HDL at baseline	2.60 ± 0.89	2.45 ± 0.77	0.480
Transfer of EC to HDL at follow-up	2.75 ± 0.73	2.73 ± 0.54	0.863
*p*-value *	0.32	0.034	
Transfer of UC to HDL at baseline	4.17 ± 1.35	3.99 ± 1.05	0.559
Transfer of UC to HDL at follow-up	4.45 ± 1.22	4.34 ± 0.89	0.678
*p*-value *	0.18	0.053	
Difference follow-up—baseline for EC	0.15 ± 0.84	0.27 ± 0.69	0.533
Difference follow-up—baseline for UC	0.28 ± 1.14	0.35 ± 0.96	0.804

* *p*-value for the comparison between baseline and follow-up. All values mean ± SD.

**Table 4:** The role of exercise on HDL transfer as categorical variables.

	Control Group (N = 31)	Exercise Group (N = 31)	*p*-Value	OR (95% CI)	*p*-int
EC above the mean at baseline (%)	48.4	41.9	0.610	0.77 (0.28–2.10)	0.783
EC above the mean at follow-up (%)	58.1	61.3	0.796	1.44 (0.41–3.16)	
*p*-value *	0.366	0.083			
UC above the mean at baseline (%)	87.1	87.1	1.000	1.00 (0.23–4.41)	NA
UC above the mean at follow-up (%)	96.8	96.8	1.000	1.00 (0.06–16.74)	
*p*-value *	0.083	0.058			

* *p*-value for the comparison between baseline and follow-up. EC; esterified cholesterol; UC: unesterified cholesterol; *p*-int: *p* for interaction.

**Table 5:** The role of exercise on lipid parameters.

Variables	Control Group (N = 31)	Exercise Group (N = 31)	*p*-Value
HDL–cholesterol at baseline (mg/dL) *	38 ± 10	38 ± 8	0.891
HDL–cholesterol at follow-up (mg/dL) *	40 ± 10	42 ± 10	0.713
Difference baseline minus follow-up *	0.92 ± 9.43	4.54 ± 6.70	0.109
*p*-value ^+^	<0.001	<0.001	
LDL–cholesterol at baseline (mg/dL) *	66 ± 26	71 ± 24	0.422
LDL–cholesterol at follow-up (mg/dL) *	79 ± 34	76 ± 27	0.634
Difference baseline minus follow-up *	46.63 ± 53.05	59.93 ± 42.99	0.306
*p*-value ^+^	0.017	<0.001	
Total cholesterol at baseline (mg/dL) *	129 ± 30	135 ± 30	0.373
Total cholesterol at follow-up (mg/dL) *	148 ± 39	141 ± 32	0.488
Difference baseline minus follow-up *	67.37 ± 65.11	69.78 ± 48.70	0.876
*p*-value ^+^	0.016	0.138	
Triglycerides at baseline (mg/dL) ^⟂^	94 (75–150)	98 (78–168)	0.720
Triglycerides at follow-up (mg/dL) ^⟂^	120 (74–201)	103 (71–149)	0.499
Difference baseline minus follow-up ^⟂^	52 (7–139)	18.5 (−23.5–61)	0.046
*p*-value ^+^	0.276	0.806	

^+^* p*-value for the comparison between baseline and follow-up. * Mean ± SD; **^⟂^** Median (25th-75th percentiles).

**Table 6:** The role of exercise on inflammation and maximal oxygen consumption.

Variables	Control Group (N = 31)	Exercise Group (N = 31)	*p*-Value
CRP at baseline ^⟂^ (mg/dL)	1.80 (0.64–3.33)	1.26 (0.46–3.99)	0.607
CRP at follow-up ^⟂^ (mg/dL)	1.58 (0.74–2.91)	1.00 (0.35–4.04)	0.184
*p*-value ^+^	0.719	0.060	
Peak VO_2_ at baseline ^⟂^ (mL·kg^−1^·min^−1^)	20.00 (16.75–24.55)	22.1 (17.20–26.20)	0.459
Peak VO_2_ at follow-up * (mL·kg^−1^·min^−1^)	21.58 ± 4.98	24.25 ± 6.76	0.092
*p*-value ^+^	0.176	0.005	

* Mean ± SD; ^⟂^ Median (25th–75th percentile). ^+^ *p*-value for the comparison of baseline and follow-up.

## Data Availability

Raw data related to this article can be made available by the corresponding author on request.
